# Ship Ranging Method in Lake Areas Based on Binocular Vision

**DOI:** 10.3390/s25206477

**Published:** 2025-10-20

**Authors:** Tengwen Zhang, Xin Liu, Mingzhi Shao, Yuhan Sun, Qingfa Zhang

**Affiliations:** 1 School of Ship and Port Engineering, Shandong Jiaotong University, Weihai 264209, China; 2 Weihai Institute of Marine Information Science and Technology, Weihai 264200, China; 3 Shandong Premium Boating Technology Ltd., Weihai 264200, China

**Keywords:** binocular ranging, ORB algorithm, stereo vision, vessel monitoring, camera calibration

## Abstract

The unique hollowed-out catamaran hulls and complex environmental conditions in lake areas hinder traditional ranging algorithms (combining target detection and stereo matching) from accurately obtaining depth information near the center of ships. This not only impairs the navigation of electric tourist boats but also leads to high computing resource consumption. To address this issue, this study proposes a ranging method integrating improved ORB (Oriented FAST and Rotated BRIEF) with stereo vision technology. Combined with traditional optimization techniques, the proposed method calculates target distance and angle based on the triangulation principle, providing a rough alternative solution for the “gap period” of stereo matching-based ranging. The method proceeds as follows: first, it acquires ORB feature points with relatively uniform global distribution from preprocessed binocular images via a local feature weighting approach; second, it further refines feature points within the ROI (Region of Interest) using a quadtree structure; third, it enhances matching accuracy by integrating the FLANN (Fast Library for Approximate Nearest Neighbors) and PROSAC (Progressive Sample Consensus) algorithms; finally, it applies the screened matching point pairs to the triangulation method to obtain the position and distance of the target ship. Experimental results show that the proposed algorithm improves processing speed by 6.5% compared with the ORB-PROSAC algorithm. Under ideal conditions, the ranging errors at 10m and 20m are 2.25% and 5.56%, respectively. This method can partially compensate for the shortcomings of stereo matching in ranging under the specified lake area scenario.

## 1. Introduction

In recent years, electric intelligent tourist boats have been increasingly applied in water-based leisure scenarios such as lakes and rivers, making passenger safety assurance paramount. To ensure the safe navigation of these vessels, effective identification of water surface obstacles and implementation of collision avoidance measures are essential. Among existing ranging technologies, the binocular stereo vision system—a non-contact ranging technique—has shown significant advantages in multiple fields owing to its cost-effectiveness and practicality [1].

The working principle of this system is as follows: two cameras capture images of the same scene from different perspectives; the system then calculates key information (e.g., the 3D coordinates of objects) using disparity, thereby obtaining accurate distance data. This process is not restricted by an object’s type, shape, or color, giving it broad adaptability. Compared with high-precision sensors such as LiDAR, the binocular vision system not only has lower hardware costs and the ability to classify targets but also can significantly reduce computing resource consumption—while ensuring ranging accuracy—through in-depth algorithm optimization and acceleration via dedicated computing resources. This makes it well-suited to meet the practical needs of low-cost application environments [2].

Mainstream algorithms typically combine binocular vision with stereo matching to generate disparity maps and calculate depth information. Among these, SGM (Semi-Global Matching) integrates the advantages of local and global algorithms: it maintains high accuracy while having relatively low computational complexity, making it widely used in the ranging field.

Wu Hongchen et al. [3] improved the SGBM algorithm by integrating a regional superposition segmentation strategy with multi-threading technology. They proposed a system self-calibration method for area measurement, which enables 3D measurement of the internal structure of workpieces by incorporating spatial Euclidean distance calculation—greatly enhancing operational speed. However, since stereo matching performs feature extraction on the entire image, it incurs large computational loads; additionally, the matching process is easily affected by noise. As a result, there is still room for improvement in real-time performance and stability.

With the development of deep learning, models can autonomously learn stereo matching patterns from images, enabling more accurate disparity calculation. Hu Qing et al. [4] proposed a novel underwater vehicle ranging system based on semantic segmentation and binocular vision. This system uses Deeplabv3+ to identify underwater targets captured by binocular cameras and generate target maps, which helps reduce ranging errors. Zhang et al. [5] proposed a stereo matching network integrating multi-scale feature attention. The network adopts an improved pyramid pooling module to enhance the extraction of effective feature information in edge regions; it also incorporates an attention module for multi-scale feature fusion. By fusing multi-scale cost volumes and the attention mechanism, the network strengthens the information content at different levels in the cost volume, thereby improving stereo matching accuracy. However, deep learning methods typically require large amounts of training data and powerful computing resources. Moreover, these models have relatively poor interpretability, which partially limits their wide application.

In scenarios with a wide variety of targets and complex backgrounds, introducing target detection algorithms enables stereo matching to be performed only on ROIs (Region of Interest). By using the depth value at the center of the matched region as the target’s overall depth, the program’s computational load can be reduced and its accuracy improved. He et al. [6] designed a water surface garbage recognition and localization system that combines YOLOv8 with binocular ranging technology. The system uses YOLOv8 to recognize garbage and the SGBM algorithm to achieve accurate ranging—significantly enhancing the recognition accuracy and localization precision of automatic recovery vessels for outdoor water garbage. Zheng et al. [7] proposed a long-distance localization scheme based on binocular vision: the scheme uses the YOLOv8 algorithm to detect maritime targets and calculates target distance according to the binocular vision principle, based on local feature extraction and matching results.

Despite significant progress in binocular ranging algorithms, they still face certain limitations in practical application scenarios. Taking the autonomous navigation of electric tourist boats in lake areas as an example, the key limitations are as follows:

(1) The coupled effect of waves and wind causes attitude disturbances in tourist boats, which may lead to the drift of camera parameters. Additionally, water surface reflection interference easily results in false matching and the loss of feature points [8].

(2) Deep learning algorithms rely on high-performance hardware, making them incompatible with the requirements of low cost, real-time performance, and simple structure for navigation and collision avoidance systems in scenic areas. Currently, state-of-the-art methods for high-precision binocular matching—such as LoFTR—exhibit excellent matching accuracy in low-texture scenarios. However, their real-time performance depends on high-performance GPUs: when processing 640 × 480 image pairs on an RTX 2080Ti GPU, the speed is 116 ms per frame [9].

(3) When catamaran hulls have hollowed-out structures or lack continuous solid textures in key regions, algorithms struggle to obtain accurate depth information in the central area of the vessels.

(4) Complex lake area scenarios significantly increase the computational load of deep learning. For edge devices with low computing power, their inference speed decreases under high loads; moreover, severe resource competition occurs during multi-task concurrency [10].

Considering the above scenario characteristics, the practical application limitations of global matching algorithms, and the fact that tourist boats maintain a certain buffer distance during collision avoidance (resulting in non-stringent requirements for ranging accuracy), this study proposes the following supplementary approach: Given that global matching algorithms can obtain valid depth information for most regions, when issues such as insufficient texture and dynamic interference near the hull center make it difficult for global matching to acquire valid depth information (and further optimizing global matching to obtain such information is highly challenging), local matching algorithms can be used to focus on distinct feature points within these regions. This enables rapid acquisition of depth reference results for the regions, which can replace the missing or low-accuracy depth information from global matching in these specific areas. Therefore, the subsequent content will focus on analyzing the principle design of the local matching algorithm and its performance verification in supplementary scenarios. Detailed discussions on aspects such as the ranging performance of global matching algorithms will be omitted.

Local matching algorithms are mainly divided into region-based matching and feature-based matching. Compared with region-based matching algorithms, feature matching algorithms offer significantly improved computational efficiency and robustness, and have been applied to some extent in the field of binocular ranging. Hua et al. [11] proposed a binocular ranging method based on ORB features and RANSAC (Random Sample Consensus). This method accelerates stereo matching through iterative pre-inspection and improves ranging accuracy using quadric surface fitting. Li et al. [12] proposed an ORB-based infrared binocular ranging method that integrates adaptive geometric constraints and the RANSAC method. It selects corresponding thresholds based on the slope and distance of initial matching point pairs, and constructs geometric constraints using slope and distance to eliminate false matching point pairs. Du Gen et al. [13] proposed an improved image matching algorithm that combines the quadtree method and the PROSAC method, which improves the mapping accuracy of visual SLAM. Tyszkiewicz et al. [14] proposed DISK—a local feature detection and matching algorithm based on reinforcement learning. Through a matching reward-punishment mechanism, it reduces false matches, and its feature point coverage rate in low-texture regions is 30% higher than that of the traditional SIFT algorithm.

Among commonly used feature matching algorithms, the ORB algorithm exhibits lower matching accuracy than SURF (Speeded Up Robust Features) and SIFT (Scale Invariant Feature Transform) but offers distinct advantages in computational efficiency and real-time performance [15]. The ORB algorithm integrates FAST (Features from Accelerated Segment Test) keypoint detection with the BRIEF (Binary Robust Independent Elementary Features) descriptor. This integration effectively speeds up feature extraction while maintaining excellent rotation and scale invariance—making it suitable for real-time scenarios such as lake area ship ranging. This study aims to use depth measurements from several distinct feature point pairs on tourist boats to estimate the overall depth of the boats. The specific process is as follows: first, YOLOv8 detects oncoming ships; second, the ORB feature matching method is applied, with uniform FAST corner points obtained via a local feature weighting approach; third, quadtree, FLANN, and PROSAC algorithms are introduced to enhance sub-pixel matching accuracy in ROIs; finally, a stereo vision-based method calculates the distance and position of the tourist boats, with results intuitively displayed on a map. Ultimately, while ensuring measurement accuracy, computing resource consumption is minimized to improve the system’s overall cost-effectiveness and practicality.

## 2. Stereo Vision Ranging Algorithm

### 2.1. Binocular Vision Principle

**Figure 1:**
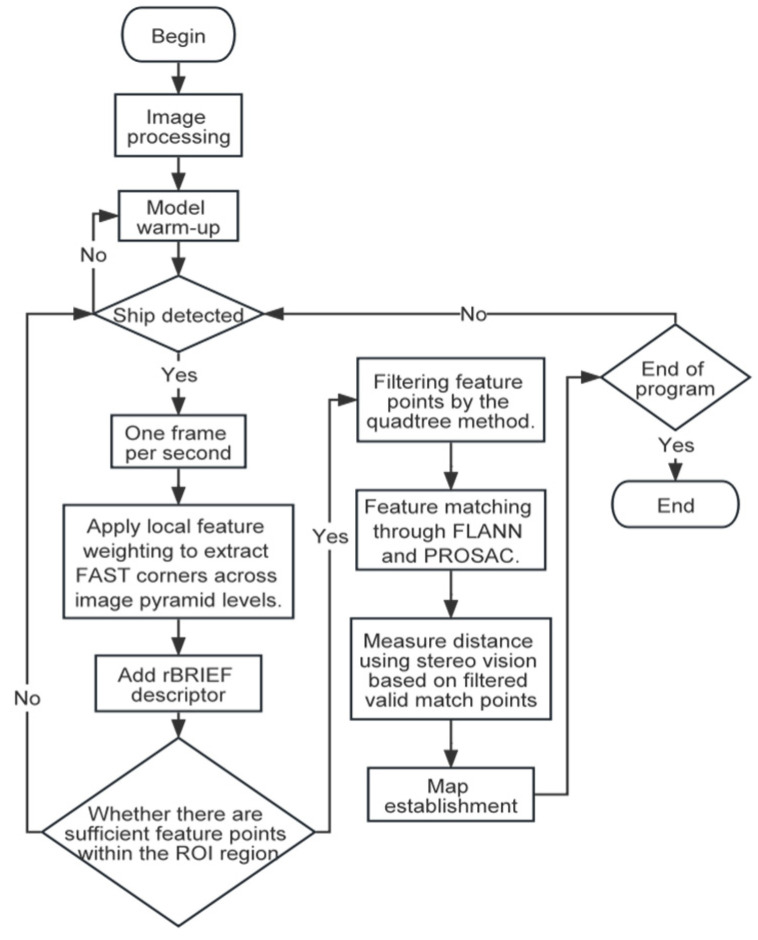
Algorithmic flow.

In this study, a binocular camera is used to acquire information about the target ship, and the specific process is illustrated in Figure 1.

**Figure 2:**
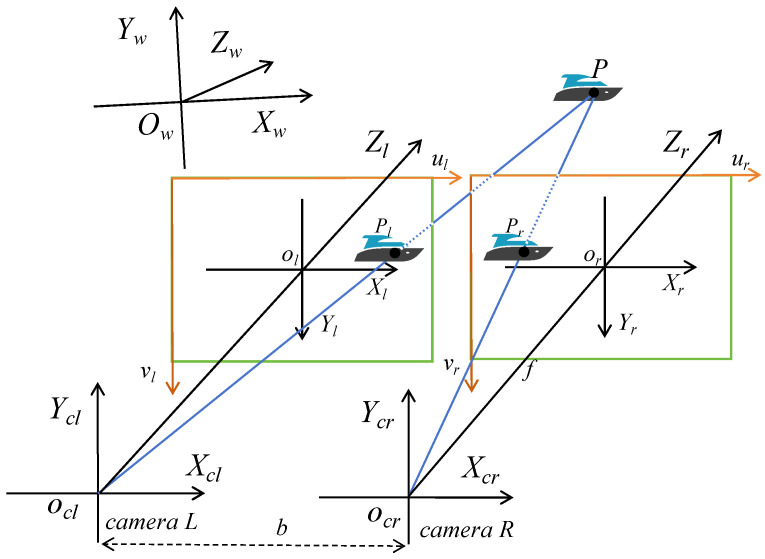
Binocular Vision Principle.

The binocular vision system is based on bionic principles, mimicking the stereoscopic vision mechanism of human eyes to measure the target depth in 3D space. It consists of two cameras with a known relative positional relationship: the binocular cameras capture the same scene from different viewing angles and project points in 3D space onto the 2D image plane using the principle of similar triangles. By matching the left and right images, the disparity of corresponding pixel points can be obtained. As shown in Figure 2, the binocular cameras (with a fixed baseline distance) capture the same scene from different perspectives. For any point P in the real world, its projections on the virtual imaging planes of the left and right cameras are denoted as $P_{l}$ and ${\;P}_{r}$, respectively; after processing, the projections only differ in the horizontal coordinate (i.e., $u_{l}$ and $u_{r}$), and the relative relationship of the pixel points can be represented by the difference. Based on the similarity of triangles, the 3D world depth of this point can be derived as shown in Equation (1).

(1) $$Z=\frac{f*b}{\left(u_{l}-u_{r}\right)}$$

where f is the focal length of the camera, b is the baseline distance between the two cameras, $u_{l}-u_{r}$ is the disparity, which is the difference in pixel coordinates between $P_{l}$ and $P_{r}\;$. The farther the actual distance, the smaller the parallax, and the more sensitive the corresponding depth value changes.

### 2.2. Coordinate System Transformation

The mapping of real-world objects to the camera imaging plane involves a series of transformations. To accurately describe and process these geometric relationships, multiple coordinate systems must be defined to ensure the accuracy of data conversion and calculations. The specific definitions are as follows:

(1) Pixel Coordinate System (PCS): This system corresponds to the grid structure of digital images, with pixels as the unit. Its origin is located at the top-left corner of the image; the horizontal direction to the right is defined as the positive direction of the u-axis, and the vertical direction downward as the positive direction of the v-axis.

(2) Image Coordinate System (ICS): This system represents the projection of objects onto the imaging plane, with meters or millimeters as the unit. Its origin is at the center of the image, and the *x*-axis and *y*-axis are parallel to the u-axis and v-axis of the PCS, respectively.

(3) Camera Coordinate System (CCS): Serving as a link between the 3D world and 2D images, this system moves with the camera, using meters or millimeters as the unit. Its origin is the optical center of the camera; the *x*-axis and *y*-axis are parallel to the image plane, while the *z*-axis (optical axis) points to the scene depth, forming a 3D Cartesian coordinate system.

(4) World Coordinate System (WCS): As a global reference framework, it describes the absolute position and orientation of objects in the environment. The selection of its origin and the directions of the 3D axes depends on specific application requirements, with meters or millimeters as the unit.

**Figure 3:**
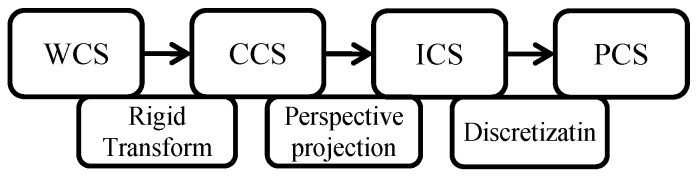
Coordinate system conversion process.

The relationships between these coordinate systems are illustrated in Figure 3. By transforming coordinates from the WCS to the CCS, then to the ICS, and finally to the PCS, the depth values of each point in the scene relative to the camera plane can be calculated.

Assume there is a point in the real world. In the WCS, this point is denoted as ${\left(X_{w},Y_{w},Z_{w}\right)}^{T}$. After rotation and translation transformations via Equation (2), its coordinate in the CCS is ${\left(X_{c},Y_{c},Z_{c}\right)}^{T}$. Here, R represents the rotation matrix of the transformation, and T represents the translation matrix of the transformation.

(2) $$\begin{array}{c} \left[\begin{array}{c} X_{c}\\ Y_{c}\\ Z_{c}\\ 1 \end{array}\right]=\left[\begin{array}{cc} R & T\\ 0 & 1 \end{array}\right]\left[\begin{array}{c} X_{w}\\ Y_{w}\\ Z_{w}\\ 1 \end{array}\right] \end{array}$$

The CCS projects points via an intrinsic parameter matrix. With reference to Equation (3), 3D points in space are converted into 2D points ${\left(X,Y\right)}^{T}$ in the ICS.

(3) $$\begin{array}{c} Z_{c}\left[\begin{array}{c} X\\ Y\\ 1 \end{array}\right]=\left[\begin{array}{cccc} f & 0 & 0 & 0\\ 0 & f & 0 & 0\\ 0 & 0 & 1 & 0 \end{array}\right]\left[\begin{array}{c} X_{c}\\ Y_{c}\\ Z_{c}\\ 1 \end{array}\right] \end{array}$$

The conversion from the ICS to the PCS is achieved by converting points represented in physical units into points in pixel units through unit conversion and origin offset. Here dx and dy denote the physical sizes of a single pixel along the *x*-axis and *y*-axis, respectively, while $u_{0}$ and $v_{0}$ represents the offset required to shift the origin to the top-left corner (of the image).

(4) $$Z_{c}\left[\begin{array}{c} u\\ v\\ 1 \end{array}\right]=\left[\begin{array}{ccc} \frac{1}{dx} & 0 & u_{0}\\ 0 & \frac{1}{dy} & v_{0}\\ 0 & 0 & 1 \end{array}\right]\left[\begin{array}{c} X\\ Y\\ 1 \end{array}\right]$$

Combining the above Equations yields the complete transformation Equations from the WCS to the PCS as given in Equation (5):

(5) $$\begin{array}{c} Z_{c}\left[\begin{array}{c} u\\ v\\ 1 \end{array}\right]=\left[\begin{array}{cccc} \frac{1}{dx} & 0 & u_{0} & 0\\ 0 & \frac{1}{dy} & v_{0} & 0\\ 0 & 0 & 1 & 0 \end{array}\right]\left[\begin{array}{cc} R & T\\ 0 & 1 \end{array}\right]\left[\begin{array}{c} X_{w}\\ Y_{w}\\ Z_{w}\\ 1 \end{array}\right] \end{array}$$

By adjusting the intrinsic and extrinsic parameter matrices of the camera, any point in the real 3D space can be accurately mapped from the WCS to the PCS.

### 2.3. Camera Correction

The initial performance of a binocular ranging system depends on the accurate calibration of camera parameters. An uncalibrated camera may introduce errors in disparity calculation, thereby reducing the accuracy of distance estimation. Meanwhile, the inherent distortion of the lens distorts the geometric relationships in images, making depth information extraction based on these images unreliable. Camera calibration and rectification are critical steps in a binocular vision system: they eliminate image distortion caused by lens manufacturing defects and improper installation positions, thereby improving the accuracy of subsequent processing. The parameters obtained through calibration mainly include intrinsic and extrinsic parameter matrices, as well as distortion parameters. Among these, the distortion parameter models are shown in Equations (6) and (7):

(6) $$\begin{array}{c} \left\{\begin{array}{ll} x_{r}=x\left(1+k_{1}r^{2}+k_{2}r^{4}+k_{3}r^{6}\right) & \\ y_{r}=y\left(1+k_{1}r^{2}+k_{2}r^{4}+k_{3}r^{6}\right) & \end{array}\right. \end{array}$$

(7) $$\begin{array}{c} \left\{\begin{array}{ll} x_{t}=x+\left[2p_{1}xy+p_{2}\left(r^{2}+2x^{2}\right)\right] & \\ y_{t}=y+\left[p_{1}\left(r^{2}+2y^{2}\right)+2p_{2}xy\right] & \end{array}\right. \end{array}$$

where (x,y) is the coordinate of any pixel, $r=\sqrt{x^{2}+y^{2}}$ represents the Euclidean distance from a pixel to the center of the image, $k_{1},k_{2}$ and $k_{3}$ are radial distortion coefficients, $p_{1}$ and $p_{2}$ are tangential distortion coefficients.

**Figure 4:**
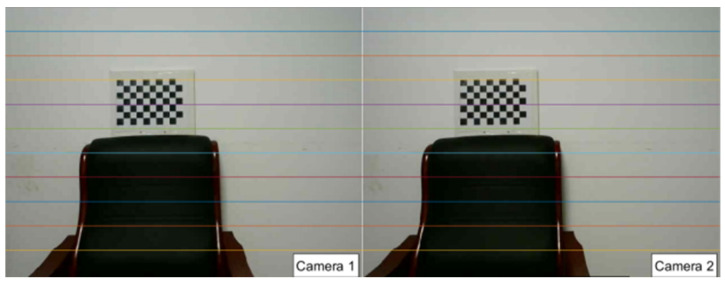
Checkerboard calibration method.

In the field of camera calibration, common methods include camera self-calibration, active vision camera calibration, and target-based calibration. Camera self-calibration typically exhibits low accuracy, as it relies on assumptions about scene structure and motion. Active vision camera calibration requires specific hardware support and complex operational procedures, leading to high implementation costs and expensive equipment. Given the actual requirements and resource constraints of the lake area ranging system, this study adopts Zhang Zhengyou’s chessboard calibration method. This method uses a chessboard pattern with known geometric properties as the calibration target. It captures multiple images from different viewing angles, automatically identifies and calculates corner coordinates via algorithms, and then accurately solves for the camera’s intrinsic and extrinsic parameters as well as distortion coefficients. As shown in Figure 4, the corresponding pixel points of the rectified binocular images lie on the same epipolar line—this simplifies the subsequent feature point matching process.

Zhang’s offline calibration method can effectively eliminate lens manufacturing and installation errors during the initial calibration phase, providing a reliable parameter foundation for the binocular ranging system. However, in complex dynamic environments such as lakes, diurnal temperature fluctuations deform camera components, leading to focal length offset and principal point drift. Meanwhile, hull vibrations disrupt the fixed baseline and relative orientation of the binocular cameras, resulting in continuous drift of offline-calibrated parameters over time; this, in turn, accumulates errors in disparity calculation. Relying solely on one-time chessboard calibration is therefore insufficient to meet the system’s stability requirements.

To address this issue, this study adopts Yin et al.’s [16] online rectification method for binocular cameras, which is highly compatible with the core technical pathway of the proposed ranging algorithm. When the tourist boat is moored, a lightweight visual SLAM (Simultaneous Localization and Mapping) system is used to construct a sparse feature point map of the dock. Optical flow tracking and grayscale cross-correlation verification are applied to optimize feature matching for binocular images; subsequently, the PnP (Perspective-n-Point) + RANSAC algorithm is used to solve for the binocular cameras’ extrinsic parameters. The optimal result is selected with epipolar rectification error as the metric, thereby compensating for parameter drift caused by temperature and vibration. If a region shows decreased feature point density, increased reprojection error, or a reduced proportion of valid matching pairs, the extrinsic parameters can be determined to have drifted, and the online rectification process is initiated.

### 2.4. Distance Measurement Algorithm

In an ideal environment with no attitude disturbance, strictly parallel camera optical axes, and only minimal pixel quantization noise, the ranging performance of triangulation and traditional disparity-based ranging algorithms—based on the same disparity input—exhibits high consistency. However, the triangulation algorithm still offers irreplaceable core advantages compared with disparity-based ranging, as shown in Table 1. This study uses the triangulation method for ranging under ideal conditions, which can lay a foundation for subsequent expansion to dynamic and complex scenarios.

**Figure 5:**
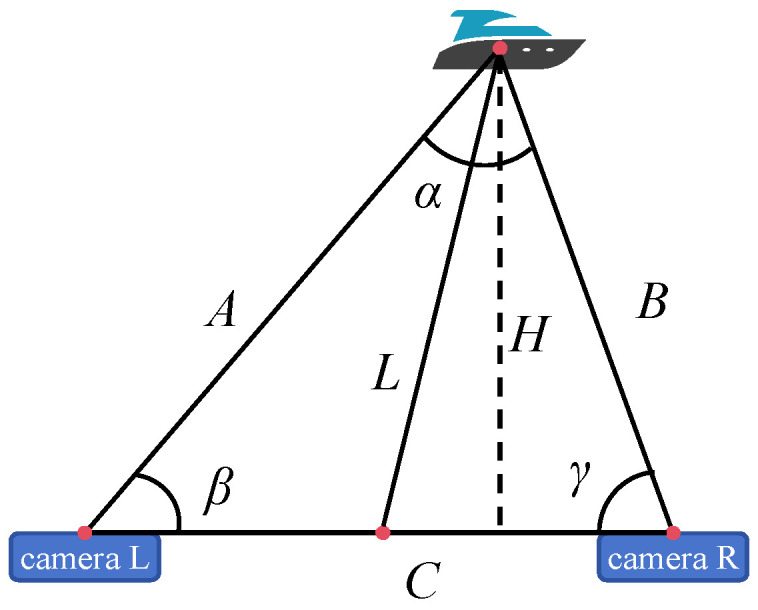
Principles of Stereoscopic Vision Measurement.

In an ideal scenario where the cameras are horizontally mounted, as shown in Figure 5, A, B and C represent the distances of the three sides of the triangle formed by the left camera, right camera, and the target ship, respectively; α, β and γ denote the angles formed by these three sides. The midpoint of the binocular camera system is regarded as the position of the ship equipped with the binocular cameras, and the straight-line distance L from this midpoint to the target ship is defined as the actual distance between the two ships. The parameter to be solved is the ideal vertical distance from the target ship to the camera frame H. Using trigonometric relationships, we can derive the following Equations:

(8) $$\begin{array}{c} H=A*sin\beta =B*sin\gamma \end{array}$$

(9) $$\frac{A}{sin\gamma }=\frac{B}{sin\beta }=\frac{C}{sin\alpha }$$

It can be inferred from Equations (8) and (9) that:

(10) $$\begin{array}{c} H=\frac{C*sin\gamma *sin\beta }{sin\alpha } \end{array}$$

Angle between target and camera plane:

(11) $$\begin{array}{c} \eta =\pi -\frac{\beta +\pi -\gamma }{2} \end{array}$$

In a binocular ranging system, calculating the ideal vertical distance H from the target ship to the camera frame requires determining the angular parameters of the triangle, where β and γ are closely related to the camera’s FOV (field of view). The FOV refers to the breadth of the scene that a camera lens can capture, a characteristic jointly influenced by lens focal length, sensor size, and shooting distance. A shorter focal length results in a wider FOV, incorporating more elements into the frame.

**Figure 6:**
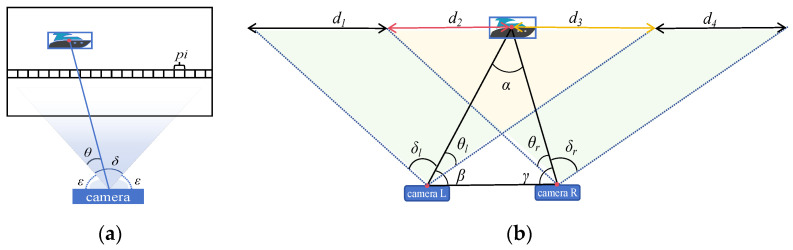
Distance calculation principle. (**a**): Relationship between camera angle and pixel; (**b**): Relationship between angle and distance calculation.

The horizontal pixel arrangement in the image maps the horizontal distribution of objects in the camera’s field of view; when the world coordinates of a target object change, its pixel position in the image shifts accordingly. For a rectified camera frame, the angle corresponding to a single pixel is determined jointly by the FOV and resolution. As shown in Figure 6, δ denotes the horizontal FOV of the camera, and θ represents the angle of the target from the edge of the imaging plane. Herein, $\varepsilon_{l}+\theta_{l}=\beta$*,*δ+2∗ε=π*,*$\varepsilon_{r}+\theta_{r}=\gamma$.${\;d}_{1}+d_{2}+d_{3}$ signifies the total number of horizontal pixels on the imaging plane corresponding to the left camera, and $d_{2}+d_{3}+d_{4}$ indicates the same for the right camera. Furthermore,${\;d}_{2}$ represents the number of pixels from the left edge of the right imaging plane to the projection of the target ship, corresponding to angle $\theta_{r}$. Conversely, $d_{3}$ denotes the number of pixels from the right edge of the left imaging plane to the projection of the target ship, corresponding to angle $\theta_{l}$. Combining Figure 5 and Figure 6, we can derive:

(12) $$\begin{array}{c} \frac{d_{3}}{\theta_{l}}=\frac{d_{1}+d_{2}+d_{3}}{\delta_{l}} \end{array}$$

(13) $$\frac{d_{2}}{\theta_{r}}=\frac{d_{2}+d_{3}+d_{4}}{\delta_{r}}$$

Combining the above Equations, Equation (14) can be obtained:

(14) $$\begin{array}{c} H=C*\frac{sin\left(\frac{d_{2}*\delta_{r}}{d_{2}+d_{3}+d_{4}}+\varepsilon_{r}\right)*sin\left(\frac{d_{3}*\delta_{l}}{d_{1}+d_{2}+d_{3}}+\varepsilon_{l}\right)}{sin\left(\pi -\frac{d_{2}*\delta_{r}}{d_{2}+d_{3}+d_{4}}-\varepsilon_{r}-\frac{d_{3}*\delta_{l}}{d_{1}+d_{2}+d_{3}}-\varepsilon_{l}\right)} \end{array}$$

where C represents the baseline of the binocular camera and δ,ε denotes the camera parameter, both of which can be obtained through program calculation.

**Figure 7:**
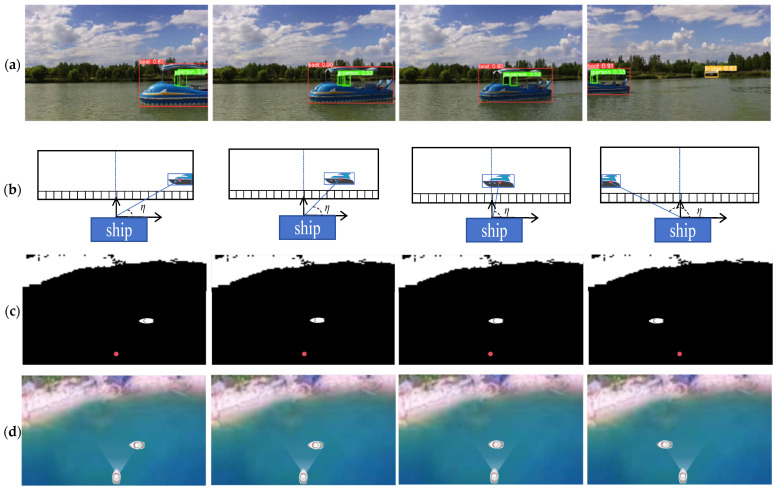
Map creation process. (**a**): Object detection; (**b**): Angle and distance calculation of PCS; (**c**): Generate white obstacles in the grid map, with red dots representing the position of the ship; (**d**): Generate user interface map.

The relative position of the target ship and the current ship can be obtained through H and η, and a visual map can be established. The process is shown in Figure 7, which can provide a basis for autonomous navigation and obstacle avoidance for cruise ships.

## 3. Image Processing and Target Detection

**Figure 8:**
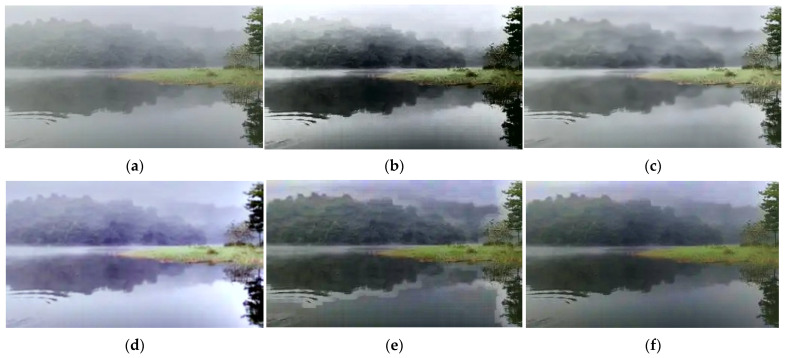
Comparison of defogging effects: (**a**) Origin; (**b**) Histogram Equalization; (**c**) CLAHE; (**d**) CLAHE; (**e**) Color Adaptive; (**f**) Multi-channel Collaboration.

**Figure 9:**
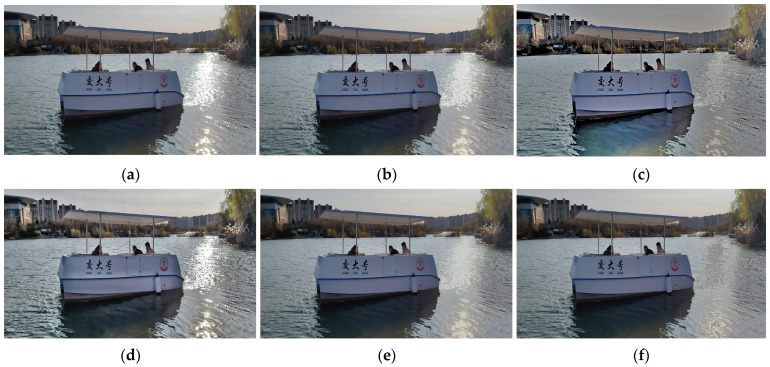
Comparison of De-Glare Algorithms: (**a**) Origin; (**b**) Threshold; Segmentation; (**c**) Retinex; (**d**) CLAHE; (**e**) Color Adaptive; (**f**) Multi-channel Collaboration.

The binocular vision ranging method proposed in this study is primarily designed to meet the autonomous navigation and collision avoidance needs of intelligent catamarans in lake areas and gentle river environments. This scenario is characterized by calm or slightly rippled water surfaces, small-to-medium-sized catamarans (the research object in this study has a length ≤ 5 m, a beam ≤ 3 m, an autonomous navigation speed ≤ 6 knots, and a safety distance of 20 m), and sparsely distributed static obstacles. Applicable weather conditions include sunny days, cloudy days, and light fog; harsh weather, hull sway, and low-light nighttime scenarios—where visual sensors are easily disabled—are not considered. This method relies on the accurate identification and matching of feature points of target objects in binocular images. However, water surface reflections in lake areas and light fog can blur target contours and even cause confusion with background grayscale [17], leading to increased matching errors and reduced ranging accuracy. Therefore, this study first compares and selects preprocessing algorithms for fog interference and water surface glare interference in the scenario, as shown in Figure 8 and Figure 9 and Table 2 and Table 3:

Mainstream methods—including Histogram Equalization, CLAHE (Contrast Limited Adaptive Histogram Equalization), Retinex, DCP (Dark Channel Prior), and GF-DCP (Guided-filter Dark Channel Prior)—were compared. Verification using indicators such as PSNR (Peak Signal-to-Noise Ratio), SSIM (Structural Similarity Index Measure), and real-time performance (as shown in Table 2) demonstrates that GF-DCP exhibits superior performance in fog removal and edge detail preservation. Additionally, it only takes 0.026 s to process an image, which meets the real-time requirements of binocular ranging. Therefore, GF-DCP was ultimately selected as the fog removal preprocessing solution.

Methods including threshold segmentation, Retinex, CLAHE, Color Adaptive, and Multi-channel Collaboration were compared. The Color Adaptive method adjusts the brightness and saturation of glare regions jointly in the HSV color space, which can effectively suppress water surface reflections. Moreover, it ensures high feature consistency between the processed binocular images and exhibits excellent real-time performance—making it suitable for the requirements of subsequent feature matching. Therefore, the Color Adaptive method was ultimately selected as the glare removal preprocessing solution in this study.

The processed images still contain a large amount of background information irrelevant to ranging; therefore, target detection is a core step to ensure the system’s accuracy and efficiency. By accurately locating the ROIs of obstacles such as ships and confining matching and ranging calculations to local regions, interference from irrelevant areas can be effectively reduced.

As a representative of one-stage target detection algorithms, the YOLO series unifies target localization and classification tasks into a single convolutional neural network. It abandons the redundant processes of region proposal and feature re-extraction in two-stage detectors, achieving a significant improvement in detection speed. This study selects YOLOv8 (You Only Look Once version 8) as the target detection model, mainly based on the following technical advantages:

(1) It adopts the lightweight CSPDarknet53 backbone network and integrates a dynamic label assignment strategy. While maintaining the high efficiency of one-stage detectors, it enhances detection capability for small and occluded targets through an adaptive feature alignment mechanism.

(2) Through a multi-scale feature fusion pyramid and an adaptive anchor box mechanism, YOLOv8 can effectively address issues such as variable ship shapes and complex lighting conditions in lake area environments. Combined with the improved WIoU (Wise Intersection over Union) loss function, it alleviates the negative impact of target occlusion and scale imbalance on ROI localization.

(3) Additionally, compared with YOLOv5, YOLOv8 reduces the number of parameters by approximately 15% through model structure simplification and computational load optimization,, making it more suitable for edge computing devices equipped on scenic area catamarans.

## 4. ORB Algorithm Improvement

The ORB algorithm focuses on regions with rapid grayscale changes in images and exhibits low sensitivity to complex environments such as water ripples and reflections, enabling more stable detection of edge points of water targets. Based on this characteristic, the ORB algorithm designed in this study consists of three parts: initial detection, feature screening, and feature matching.

(1) Initial detection: First, determine whether the image contains fog or glare. If so, implement the algorithms described in Chapter 3; then, use the ORB algorithm to detect feature points in the binocular images.

(2) Feature screening: Recursively divide the image into four sub-regions using a quadtree, and screen relatively significant feature points from each sub-region to reduce feature point redundancy.

(3) Feature matching: Use the FLANN algorithm to find the closest matches between feature points of the two images, and further improve sub-pixel matching accuracy with the PROSAC method.

### 4.1. Initial Detection

**Figure 10:**
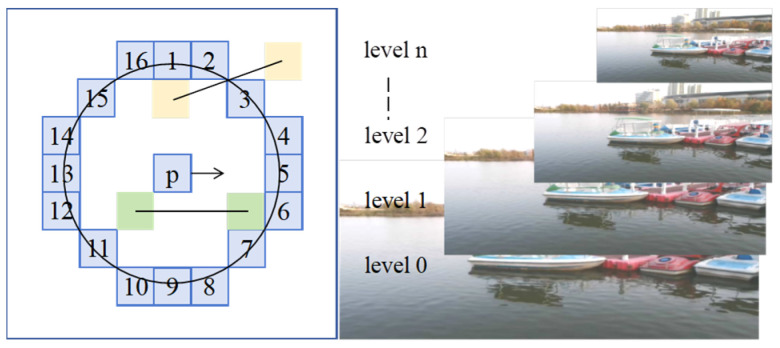
Orb algorithm schematic.

The ORB algorithm combines the efficient feature point detection capability of the FAST algorithm with the fast feature description of the BRIEF descriptor, and achieves rotation and scale invariance through an image pyramid. Compared with other feature detection algorithms, it performs better in operational speed and resource consumption, making it suitable for application scenarios requiring real-time processing [18]. The detection principle is illustrated in Figure 10. Let the pixel threshold be T and the grayscale value be L. Take any point p in the image; generally, if there are at least 12 consecutive pixel points on a circle with a radius of 3 pixels where the grayscale difference from point p satisfies:

(15) $$\begin{array}{c} \left|L_{i}-L_{p}\right|>T\;,i=1,2,\ldots ,16 \end{array}$$

where $L_{i}$ represents the grayscale value of the i-th pixel within the circular ring, $L_{p}$ is the grayscale value of the central point p.

If this condition is met, p is identified as a FAST-12 corner.A higher threshold T results in fewer detected corners.

The ORB algorithm uses a fixed threshold for FAST detection. When environmental contrast decreases, the number of detected feature points tends to drop sharply. To address this issue, this study introduces an illumination-adaptive threshold calculation method based on local grayscale statistics, which enables dynamic threshold adjustment according to the lighting conditions around candidate points.

(1) Calculate the mean grayscale value μ of a circular ring with a radius of 3 pixels, centered at the feature point to be detected.

(16) $$\mu =\frac{1}{w\times h}\sum_{w\times h}\;I\left(x,y\right)$$

where $I\left(x,y\right)$ represents the grayscale value of each pixel on the image, w denotes the pixel width, and h denotes the pixel height.

(2) Calculate the deviation between each sampling point in the neighborhood and the mean grayscale value ε, as well as the sum of squared deviations ξ.

(17) $$\varepsilon =I\left(x,y\right)-\mu$$

(18) $$\xi =\sum_{I}\;[I(x,y)-\mu {]}^{2}$$

(3) Calculate the pixel distribution probability P of the grayscale differences between 16 pixels and the mean grayscale value; the adaptive threshold is then given by Equation (20).

(19) $$P=P_{\varepsilon }(i,j)$$

(20) $$T\;=k\sum_{\varepsilon }\;P\xi$$

where *k* is a constant in [0, 1].

By correlating the threshold with brightness information, the ORB algorithm can adaptively adjust to varying lighting conditions, significantly improving detection efficiency. To further enhance precision, a local grayscale change model is fitted to refine the initially detected feature points, obtaining their sub-pixel-accurate positions. Additionally, the centroid method based on grayscale values is used to compute the orientation of each corner, yielding feature points with directional information.

The BRIEF algorithm operates by selecting n pairs of pixels within the s×s neighborhood window of a feature point and comparing their grayscale values according to Equation (21):

(21) $$\rho \left(p;x,y\right)=\left\{\begin{array}{cc} 1,\;\; & if\;L_{x}>L_{y}\\ 0,\;\; & otherwise \end{array}\right.$$

(22) $$f_{n}\left(p\right)=\sum_{1\leq i\leq n}2^{i-1}\rho \left(p;x_{i},y_{i}\right)$$

where $L_{x}$ and $L_{y}$ are the grayscale values of the paired pixels.

The comparison results are combined into a binary descriptor of a specified length—typically 128, 256, or 512 bits—as per Equation (22). To achieve rotation invariance, a rotation matrix is constructed to rotate the set of pixel pairs, aligning them with the orientation of the feature point and resulting in a rotation-invariant BRIEF descriptor. Image pyramids are utilized to scale images according to specified ratios and levels, allowing the ORB algorithm to operate on each pyramid layer. This process enables the detection of feature points across different scales, thus achieving scale invariance.

### 4.2. Feature Screening

Feature points obtained by the ORB algorithm exhibit redundancy and insufficient distribution uniformity, which may easily lead to feature point mismatching and reduce ranging accuracy. Common feature point homogenization methods include quadtree and grid partitioning:

The quadtree is a tree data structure for spatial partitioning, where the basic unit is a node representing a rectangular region. Nodes are recursively divided into four quadrants until the number of feature points in each quadrant does not exceed a preset threshold. The grid partitioning method divides the image into fixed-size grid units, retaining a specified number of feature points in each unit.

**Figure 11:**
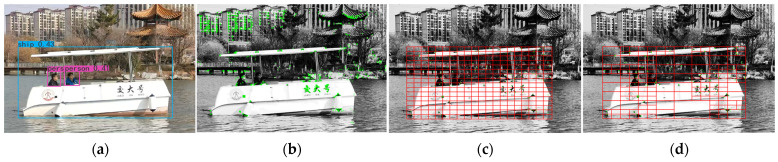
Comparison of feature point screening effect. (**a**): Generate ROI area (the blue area): (**b**): Detected feature points (the green ones); (**c**): Screen feature points by Grid partitioning method (the red area); (**d**): Screen feature points by Quadtree method (the red area).

This study uses the quadtree method to screen feature points in the ROI detected by YOLO, as shown in Figure 11. Compared with the grid partitioning method, the quadtree method offers higher flexibility and dynamic adjustment capability, with shorter processing time—making it suitable for processing complex backgrounds and multi-scale feature points.

### 4.3. Feature Matching

#### 4.3.1. Matching Based on FLANN

**Figure 12:**
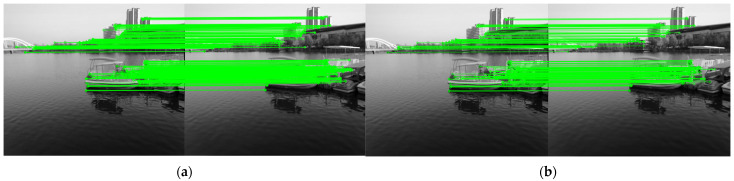
Matching effect comparison. (**a**): Matching effect with FLANN(green lines); (**b**): Matching effect with knnMatch (green lines).

Mismatching of feature points will seriously affect the calculation of target disparity, thereby impairing the accuracy of the entire system. This study first uses FLANN for initial matching. FLANN stores the Euclidean distance between two points in a KD-tree (K-Dimensional Tree); during traversal and search, each node has equal priority until the nearest matching point to the feature point is found. As shown in Figure 12, after applying the ratio test, compared with brute-force search-based knnMatch (k-nearest-neighbors match), FLANN exhibits slightly lower accuracy but significantly better performance in efficiency and resource consumption. Table 4 shows the specific parameter settings for FLANN.

#### 4.3.2. Matching Based on PROSAC

**Figure 13:**
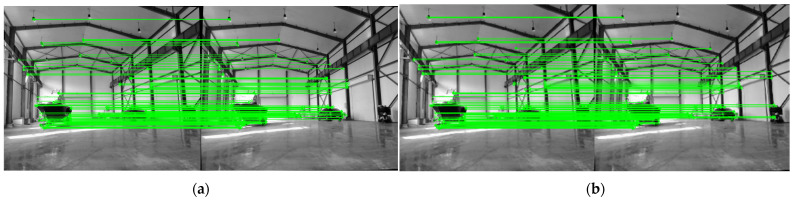
Matching effect comparison. (**a**): Matching effect with RANSAC (green lines); (**b**): Matching effect with PROSAC (green lines).

After initial matching, the PROSAC algorithm is used to further eliminate mismatched point pairs. PROSAC is an improved version of the RANSAC algorithm that incorporates a semi-random sampling strategy based on similarity ranking. It samples from a continuously expanding set of best corresponding points: in the early stage, it tends to select samples from high-density regions, and as the number of samples increases, it gradually considers samples from low-density regions. This addresses issues with RANSAC—such as high iteration counts, poor real-time performance, and unstable robustness [19]. The relevant parameters are shown in Table 5. As shown in Figure 13, compared with RANSAC (which iterates with a fixed number of samples), PROSAC can improve matching accuracy, shorten iteration time, and maintain a high number of inliers.

## 5. Discussion

**Figure 14:**
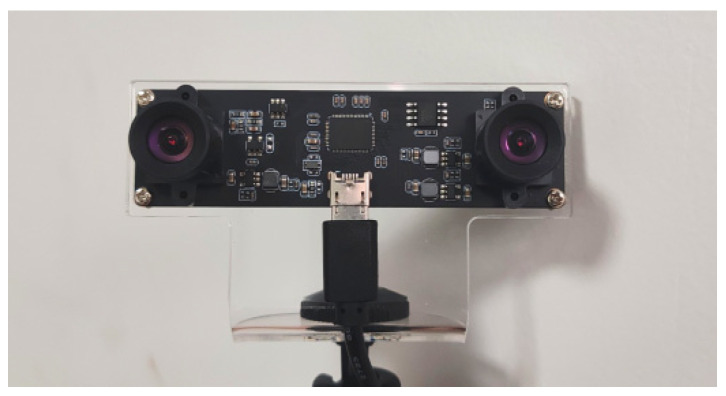
Stereo Camera Module.

All images captured in this study were acquired using a binocular camera module with 30 FPS (frames per second). The camera has a baseline of 6 cm, a focal length of 3 mm, a resolution of 480p, and a horizontal FOV of 80° for a single lens, with a theoretical effective ranging distance of 4 cm to 2200 cm. The experimental laptop is equipped with an Intel Core i5-7300HQ processor and an NVIDIA GeForce GTX 1050 graphics card. The intrinsic parameters of the camera are listed as Figure 14 and Table 6.

Considering interferences such as water surface ripples and transient illumination changes in consecutive frames—which are likely to introduce meaningless fluctuations in feature matching—and to reserve a certain amount of computing power for subsequent path planning and collision avoidance, this study designs a fixed-interval frame extraction strategy. Specifically, stable images are extracted every 0.5 s for calculation, ensuring the average frame rate of the video is stably maintained at approximately 20 FPS (the average video frame rate is 16 FPS when the SGBM algorithm is running).

**Figure 15:**
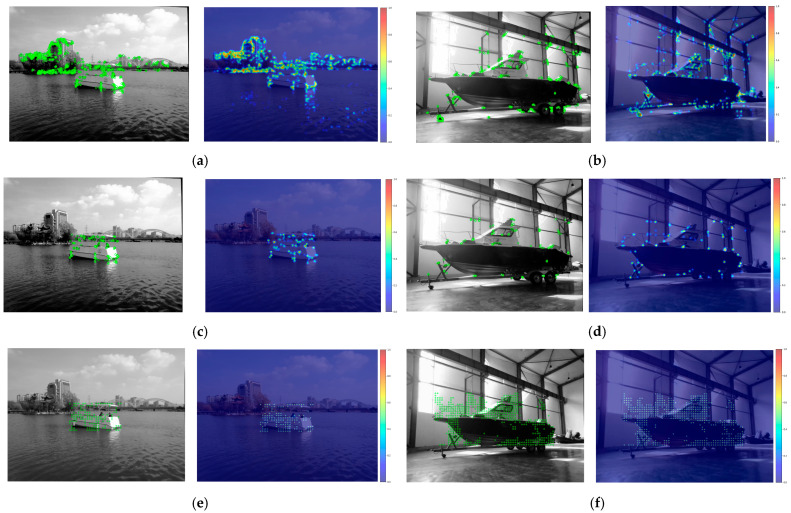
Matching Point Density Heatmaps(colored dots): (**a**) Original ORB algorithm (lake environment); (**b**) Original ORB algorithm (terrestrial environment); (**c**) Proposed algorithm in this study (lake environment); (**d**) Proposed algorithm in this study (terrestrial environment); (**e**) LoFTR algorithm (within ROI, lake environment); (**f**) LoFTR algorithm (within ROI, terrestrial environment).

**Figure 16:**
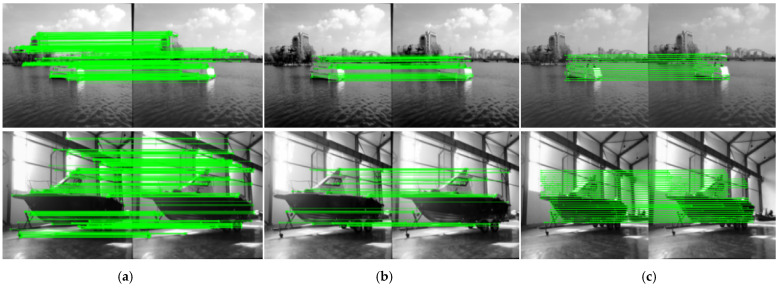
Algorithm Effect Comparison(green lines): (**a**) Detection and matching effect of ORB-PROSAC algorithm in lake and terrestrial scenes; (**b**) Detection and matching effect of our proposed algorithm in lake and terrestrial scenes; (**c**) Detection and matching effect of LoFTR algorithm in lake and terrestrial scenes.

This study selects different water-land scenarios to verify the reliability of the improved ORB algorithm, with the LoFTR dense matching algorithm added as a control. Figure 15 shows a comparison of heatmaps of different matching point densities. Although the number of matching points of the proposed algorithm is not as dense and uniform as that of the LoFTR algorithm, compared with the ORB-PROSAC algorithm, it can effectively screen feature points in the ROI and improve matching accuracy. Figure 16 presents a comparison of the matching effects of the three algorithms.

Through comparative ablation experiments, it can be concluded from Table 7 that under the same environment, the average running time of the proposed ORB algorithm for processing one frame of image (0.1392 s) is 6.5% faster than that of ORB-PROSAC (0.1489 s) and far better than that of LoFTR (2.3135 s), meeting the real-time requirement. The average matching distance of the proposed algorithm is reduced by 20.8%, 9.5%, and 6.8% compared with ORB-PROSAC, ORB-FLANN-PROSAC, and ORB-Quadtree-PROSAC, respectively, and is slightly higher than that of LoFTR.(Note: The “average matching distance” refers to the average deviation of pixel coordinates of correctly matched feature points between two images; a smaller value indicates higher matching accuracy. The “average nearest-neighbor distance” reflects the average distance between the descriptor of a feature point and that of its most similar feature, serving as an auxiliary indicator to reflect the feature discrimination ability.)

After adopting the quadtree, the average nearest-neighbor distance of the proposed algorithm is 1.65 times that of ORB-PROSAC. Although this value is higher than that of LoFTR, the uniformity of matched point pairs is significantly better than that of traditional ORB variants.

Experiments show that although the introduction of the quadtree structure and the nearest-neighbor algorithm increases complexity, the algorithm can still achieve near-real-time processing performance through the application of model preheating and parallel processing technologies [20]. Moreover, after feature point screening, the false matching rate of the proposed algorithm (3.688%) is only higher than that of ORB-PROSAC (3.242%) and LoFTR (2.193)—demonstrating balanced overall performance.

When the catamaran sails at a low speed for sightseeing, a safety distance of 20 m can meet most collision avoidance requirements. Additionally, the measurement accuracy of the binocular camera decreases as the distance increases. Given the difficulty in measuring the actual distance in the water surface environment and equipment limitations, this study adopts land-based measurement as an alternative ranging solution. Therefore, under indoor lighting conditions, 5 groups of distances within the range of 10−20 m were selected. The stereo vision ranging Equation (14) proposed in this study was used for measurement, and the ranging error was analyzed. Due to the irregular shape of the catamaran hull, the matching point pairs with the largest number of consistent disparities were selected as the ranging pixel coordinate pairs, and the closest distance from the camera to the hull was taken as the actual standard distance.

**Figure 17:**
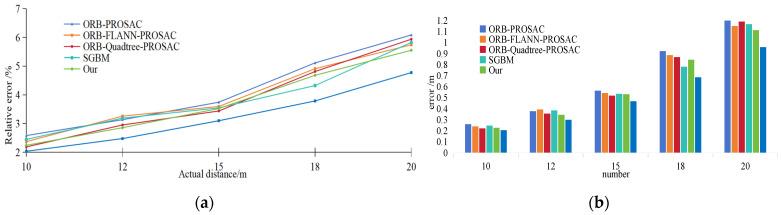
Measurement accuracy comparison. (**a**): Variation degree of ranging error of each algorithm; (**b**): Errors between algorithms and real values.

The ranging results are shown in Table 8, and Figure 17 presents a comparison of the accuracy of different ranging algorithms—indicating that the error becomes more sensitive as the distance increases. The ranging results of SGBM are calculated based on the depth mean of 5 feature points near the center of the ROI. However, the spatial positions of these 5 sampling points dynamically change with distance and the movement of the ROI center, leading to certain errors and fluctuations in the final averaged ranging results. As a deep learning-based dense matching algorithm, LoFTR can generate more numerous and spatially uniform matching points through global feature correlation. It significantly outperforms ORB-series algorithms in ranging accuracy, with a slower error growth rate. Nevertheless, it detects a large number of redundant background matching points irrelevant to ranging within the ROI; thus, the matching points used for ranging in this study are manually screened results.

Experimental data show that under ideal environments, the improved ORB algorithm in this study exhibits slow error growth within the ranging range: the relative error is 2.25% at 10 m and reaches 5.56% at 20 m. Although its accuracy is lower than that of SGBM and LoFTR, it achieves an average relative error of 3.773%—which still offers certain advantages compared with other ORB-series algorithms (ORB-FLANN-PROSAC: 3.861%, ORB-Quadtree-PROSAC: 3.937%, ORB-PROSAC: 4.152%) and meets the requirements of practical applications.

## 6. Conclusions

This study proposes a ranging method that optimizes the ORB algorithm based on traditional approaches and integrates the triangulation principle. It aims to provide a feasible alternative ranging solution when the conventional YOLOv8-SGBM algorithm fails to accurately measure the distance to target catamarans. The proposed method calculates the target’s distance and angle via trigonometric functions by measuring the target’s pixel positions in binocular images, thereby enabling representation of the target on a map.

The improved ORB algorithm follows these steps: first, it uses YOLOv8 to identify the ROI; second, it employs a quadtree structure to screen feature points in preprocessed images; third, it integrates the FLANN and PROSAC algorithms to enhance matching accuracy; and finally, it further screens suitable matching points for the ranging process to obtain the orientation and distance of the target catamaran.

Experimental comparison results indicate that the proposed algorithm meets the ranging requirements for specific lake scenarios in terms of the uniformity of acquired matching points, processing efficiency, and ranging stability. Thus, it can act as a supplementary solution for stereo ranging methods.

However, the application scope of the study’s results has certain limitations: the method is only applicable to safety distance estimation for specific types of ships in ideal environments, and no on-site tests in lake areas have been carried out, leading to insufficient environmental validation. In future research, we plan to not only upgrade the hardware (e.g., CPU, GPU) but also equip the catamaran with an IMU, edge computing devices, and a laser rangefinder. With this setup, we will verify the stability of the proposed algorithm in appropriate environments during the actual operation of tourist catamarans in lake areas and initiate research on new algorithms.

## Figures and Tables

**Table 1:** Comparison of Triangulation and Disparity-based Ranging Algorithms.

Characteristic	Triangulation	Disparity-Based Ranging
Optical Axis Constraint Dependence	Supports multi-optical-axis configurations, adaptable to non-ideal installation and attitude changes	Relies on strictly parallel optical axes
Sensitivity to Parameter Errors	Reduces the propagation efficiency of disparity errors through the geometric complementary relationship between direction angles and baseline	Ranging accuracy strictly depends on the accuracy of disparity measurement
Engineering Scalability	Can be migrated to dynamic attitude scenarios and multi-configuration binocular systems without algorithm reconstruction	When the environment or configuration deviates from the ideal state, re-design of disparity optimization and attitude calibration logic is required
System Complexity	Only requires an IMU (Inertial Measurement Unit); attitude compensation can be achieved by integrating the EKF (Extended Kalman Filter)	Requires a high-precision gimbal to maintain parallel optical axes, and relies on complex stereo matching algorithms to optimize disparity calculation
Anti-interference Capability	Can combine real-time attitude data from IMU to dynamically correct errors and offset environmental interference	Optical axis offset caused by tourist boat will be directly superimposed on disparity measurement

**Table 2:** Defogging Algorithm Metrics.

Metrics	Histogram Equalization	CLAHE	Retinex	Dark Channel Prior	Guided -Filter Dark Channel
PSNR	14.770	26.030	17.290	13.730	14.020
SSIM	0.734	0.923	0.920	0.789	0.858
Contrast	0.588	0.275	0.294	0.426	0.420
Information	7.470	7.250	7.310	7.240	7.210
Fog Density	0.458	0.553	0.607	0.352	0.355
Consumed/s	0.169	0.156	0.328	0.011	0.026

**Table 3:** Anti-Glare Algorithm Indicator.

Metrics	Threshold Segmentation	Retinex	CLAHE	Color Adaptive	Multi-Channel Collaboration
PSNR	34.99	18.02	24.44	30.11	23.09
SSIM	0.991	0.888	0.933	0.978	0.943
Contrast	0.498	0.364	0.470	0.493	0.482
Information	7.600	7.060	7.690	7.580	7.550
Consumed/s	0.094	14.612	2.892	0.094	0.578

**Table 4:** Parameter settings.

Number of Hash Tables	Hash Key Length/Bits	Multi-Probe Search Level	Number of Search Checks
12	20	2	35

**Table 5:** Parameter settings.

Reprojection Threshold/Pixel	Confidence Level for Algorithm Success	Maximum Number of Iterations
1	0.9	200

**Table 6:** Camera intrinsic parameters.

Camera	fx/Pixels	fy/Pixels	cx/Pixels	cy/Pixels	Distortion Coefficients
left_camera	520.6202	520.8697	324.7932	239.8296	k1 = −0.06858, k2 = 0.29498, p1 = −0.00789, p2 = −0.00075, k3 = −0.46509
right_camera	517.9358	517.9745	318.9745	242.9625	k1 = −0.06185, k2 = 0.26219, p1 = −0.00727, p2 = 0.00229, k3 = −0.40955

**Table 7:** Terrestrial performance index.

Model	Running Time/s	Mismatch Rate %	Average Match Distance/Pixels	Average Nearest Neighbor Distance/Pixels
ORB-PROSAC	0.1489	3.242	7.3944	1.3870
ORB-FLANN-PROSAC	0.1517	4.947	6.4709	
ORB-Quadtree-PROSAC	0.1364	7.281	6.2876	3.6841
Our	0.1392	3.688	5.8562	
LoFTR	2.3135	2.193	4.9752	2.6583

**Table 8:** Terrestrial distance measurement result.

Number	ORB -PROSAC/m	ORB-FLANN -PROSAC/m	ORB-Quadtree -PROSAC/m	SGBM/m	Our/m	LoFTR/m	Actual -Distance/m
1	10.2682	10.2371	10.2187	10.2450	10.2243	10.2035	10
2	12.3763	12.3511	12.3642	12.3826	12.3429	12.2873	12
3	15.5616	15.5194	15.5398	15.5322	15.5276	15.4649	15
4	18.9204	18.8639	18.8863	18.7784	18.8426	18.6817	18
5	21.2172	21.1488	21.1876	21.1652	21.1135	20.9553	20

## Data Availability

The original contributions presented in this study are included in the article. Further inquiries can be directed to the corresponding author(s).
